# Hydatid Cyst of the Thigh: A Report of a Rare Case With Literature Review

**DOI:** 10.7759/cureus.46040

**Published:** 2023-09-26

**Authors:** Arshadullah Khan, Renad AlSubaie, Aeshah A Aljayban

**Affiliations:** 1 Surgical Oncology and Breast Oncoplasty, Al Ahsa Hospital, Al Hofuf, SAU; 2 Medicine and Surgery, King Faisal University, Al Ahsa, SAU

**Keywords:** hydatidosis, thigh, musculoskeletal, echinococcosis, hydatid cyst

## Abstract

Hydatid cyst (HC) is a parasitic infection originating from the cestode of the *Taeniidae* family, predominantly occurring in the liver and lungs. Muscular involvement, however, remains a rarity. This report delineates the case of a 32-year-old female from Saudi Arabia presenting with a six-month history of an enlarging mass in her left thigh accompanied by significant localized pain. The clinical features were suggestive of an abscess, yet diagnostic imaging, specifically ultrasound, uncovered the presence of multiple daughter cysts within a primary lesion. MRI confirmed a well-defined encapsulated cystic mass. With an additional backdrop of a previously treated hepatic HC and living in proximity to a domestic cat, the clinical suspicion gravitated toward echinococcosis. Following surgical excision of the cystic lesion, histopathological examination confirmed the diagnosis of an infected HC. Postoperatively, the patient displayed an uneventful recovery with no recurrence or complications. This case underscores the diagnostic challenge posed by HC, especially in non-typical locations, and the pivotal role of imaging modalities in facilitating accurate diagnosis and subsequent management.

## Introduction

Hydatid cyst (HC), scientifically referred to as echinococcosis cyst, is considered to be one of the important zoonotic diseases and has significant public health importance due to the difficulties of the diagnosis [1]. It is a parasitic infection sourced from the cestode of the *Taeniidae* family [2,3]. These organisms exist in both definitive and intermediate hosts, thereby establishing a complex ecological interplay [3]. Central to the sustenance of this cycle is a transmission mechanism characterized by the propagation of the disease to intermediate hosts, which encompass a spectrum including cattle, sheep, humans, goats, horses, and camels. This transmission is facilitated by the release of eggs into the environment through the excretions of definitive hosts such as dogs, wolves, or foxes [4,5].

In general, Mediterranean countries, the Middle East, India, and Australia are the endemic areas of hydatid disease due to the prominence of animal husbandry in these regions [5].

The location of the cyst affects the clinical presentation, which differs from case to case. Years may pass without any symptoms for the patient. However, patients whose cyst mass continues to increase in size will be more likely to experience symptoms. Furthermore, cyst rupture is more susceptible to causing unexpected symptoms and signs [6].

The manifestation of HCs within the muscular tissue is an infrequent phenomenon, exhibiting an incidence ranging from 1% to 5.4% within the comprehensive spectrum of hydatid locations [7]. Remarkably, this anatomical site represents the third most prevalent localization, trailing behind pulmonary and hepatic localizations. Notably, in a subset of cases, the manifestation of splenic involvement is posited as a requisite antecedent to muscular encroachment, with an estimated prevalence of 8% [8]. Infection of primary muscle cystic echinococcosis is very rare, and only a few cases have been identified in the literature.

The clinical suspicion of echinococcosis is warranted when encountering patients originating from rural areas who exhibit progressive soft tissue mass development. Additionally, the differential diagnosis of limb masses, encompassing entities such as abscesses, both benign and malignant neoplasms, calcified hematomas, and lipomas, underscores the significance of considering echinococcosis as a prominent diagnostic contender [9]. As a proactive measure to mitigate the potential risk of anaphylactic reactions, the evaluation of an echinococcosis diagnosis is recommended prior to embarking upon a surgical biopsy [10].

Here, we report a case of thigh HC with a comprehensive discussion of its clinical presentation, diagnosis, management, and a review of relevant literature.

## Case presentation

A 32-year-old female who is residing in Saudi Arabia, specifically Al Ahsa city, presented at a general surgery clinic with a six-month history of progressively enlarging left thigh swelling. The condition recently evolved into intense localized pain, particularly exacerbated during physical activity. Remarkably absent are chronic diseases or comorbidities, with her medical history notable for a previous hepatic HC surgically managed a year prior. It is worth noting that according to the patient, she experienced swelling even before her liver HC diagnosis, suggesting a possible missed diagnosis in the past.

On examination, the patient displayed an otherwise well state with normal vital signs. A localized assessment revealed a palpable mass measuring about 6 x 7 cm on the posterior aspect of her left lower thigh, accompanied by erythema. Palpation of the mass revealed heightened temperature, tenderness, a non-fluctuant, and firm consistency, consistent with localized inflammation. Interestingly, she shared her living space with a domestic cat. This comprehensive clinical portrait aligns with characteristics suggestive of an abscess formation, warranting further diagnostic evaluation and intervention. The results of the patient’s laboratory tests were all within normal ranges.

**Figure 1:**
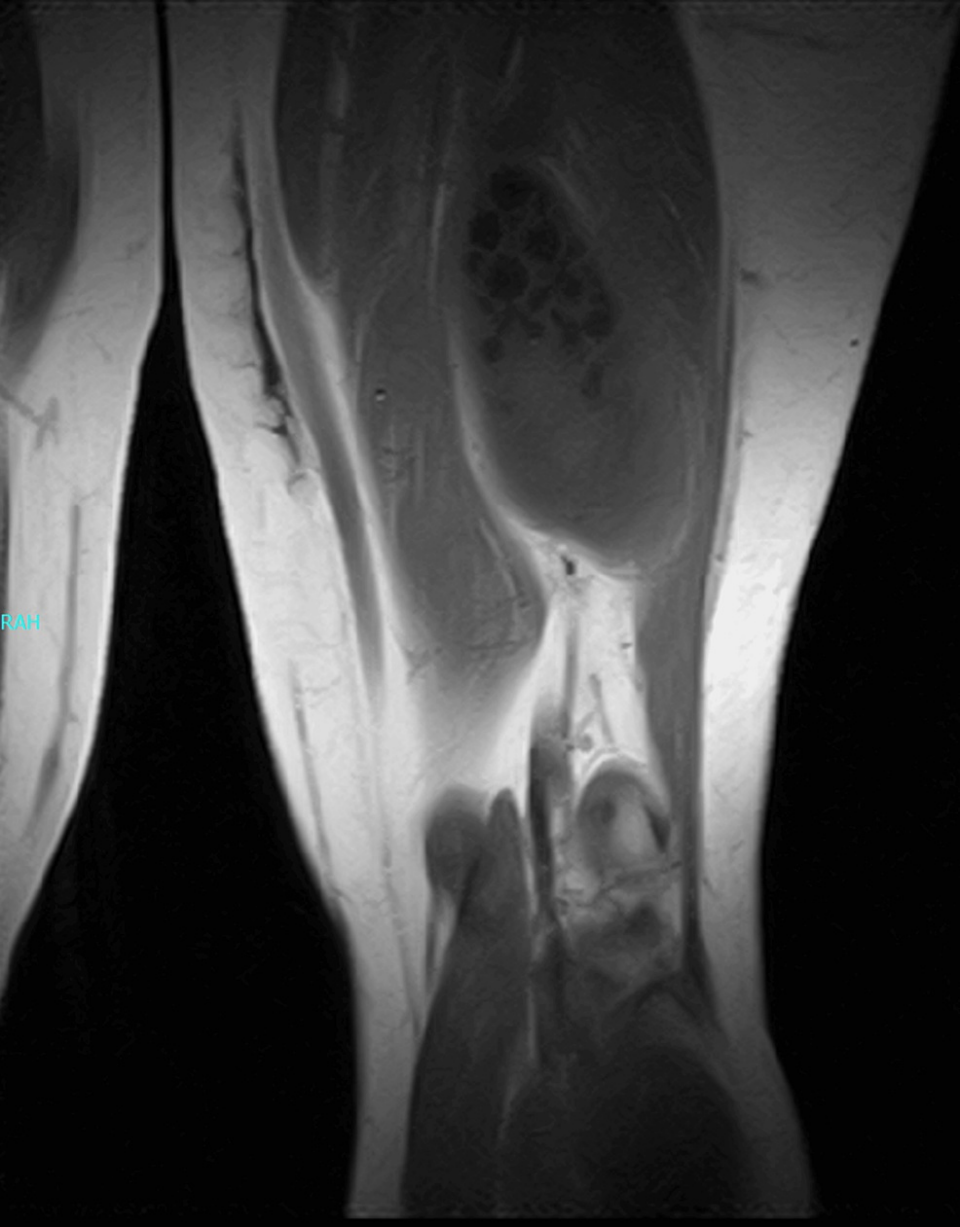
MRI of the lesion in the thigh (before surgery)

**Figure 2:**
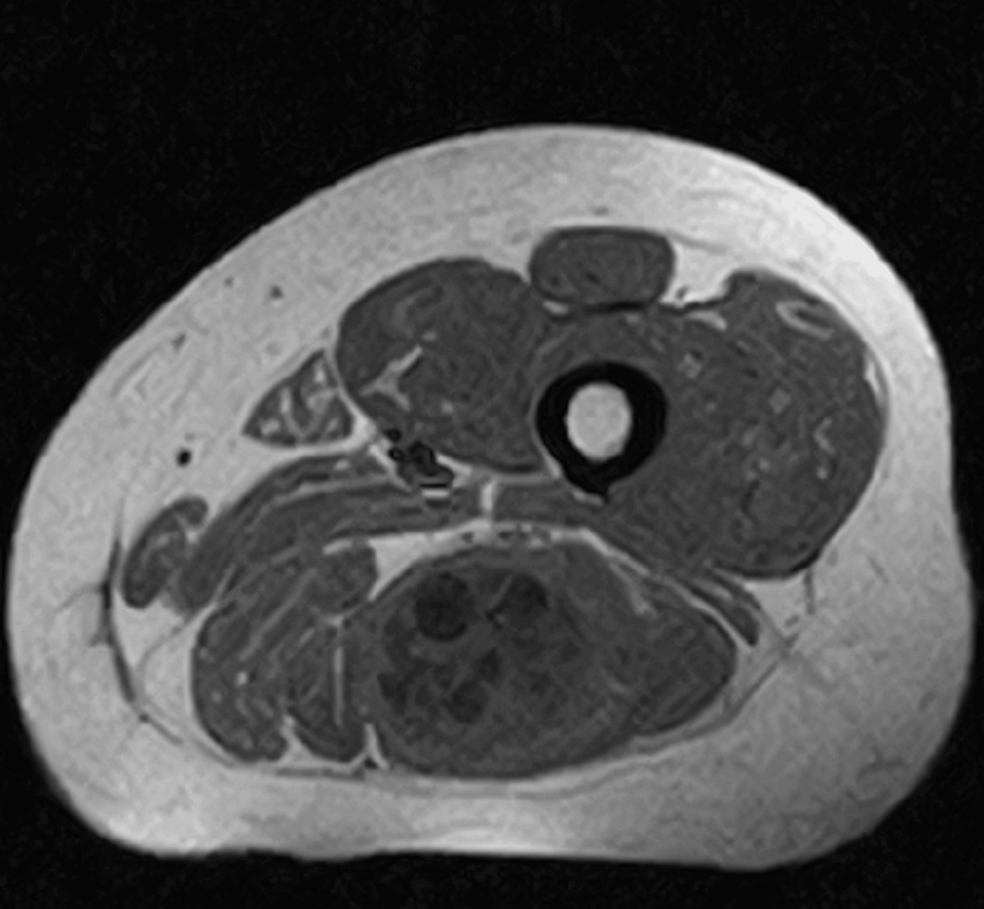
Cross-sectional MRI demonstrating the lesion in the left thigh

MRI revealed a well-defined encapsulated cystic lesion in the intramuscular region of the back of the thigh, which is considered an unusual place of spread (Figures 1, 2), while complementary ultrasound corroborated the findings, revealing multiple daughter cysts within the primary cystic lesion, indicative of a possible HC.

Intraoperatively, using spinal anesthesia, a longitudinal cut was made over the skin covering the mass on the left lower thigh. While dissecting through the muscle layers, a cystic structure, resembling an HC, was identified. Pads soaked in hypertonic saline were placed around the cyst to mitigate the risks associated with potential spillage. With utmost precision, the cyst was aspirated and then excised without causing a rupture.

**Figure 3:**
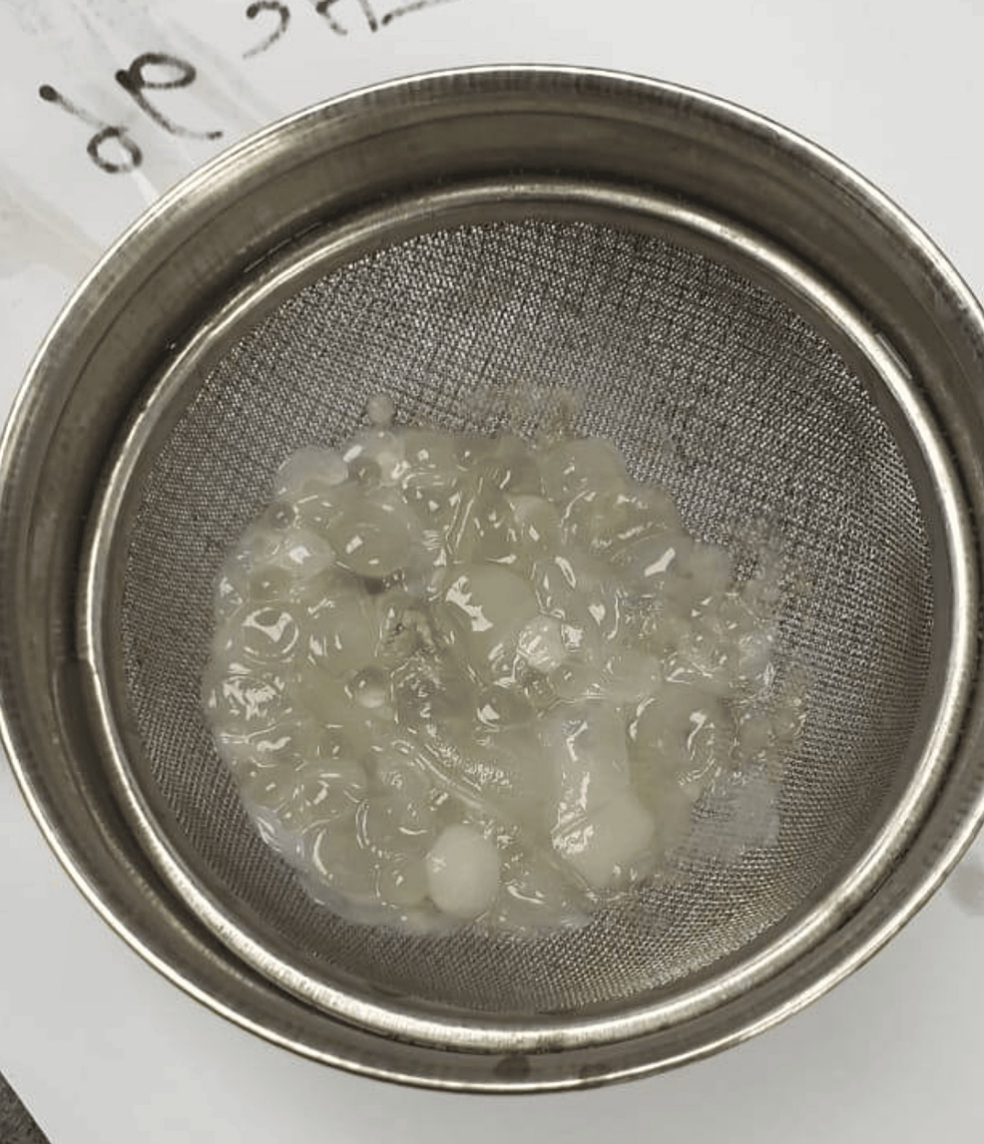
Macroscopic cyst of the thigh

**Figure 4:**
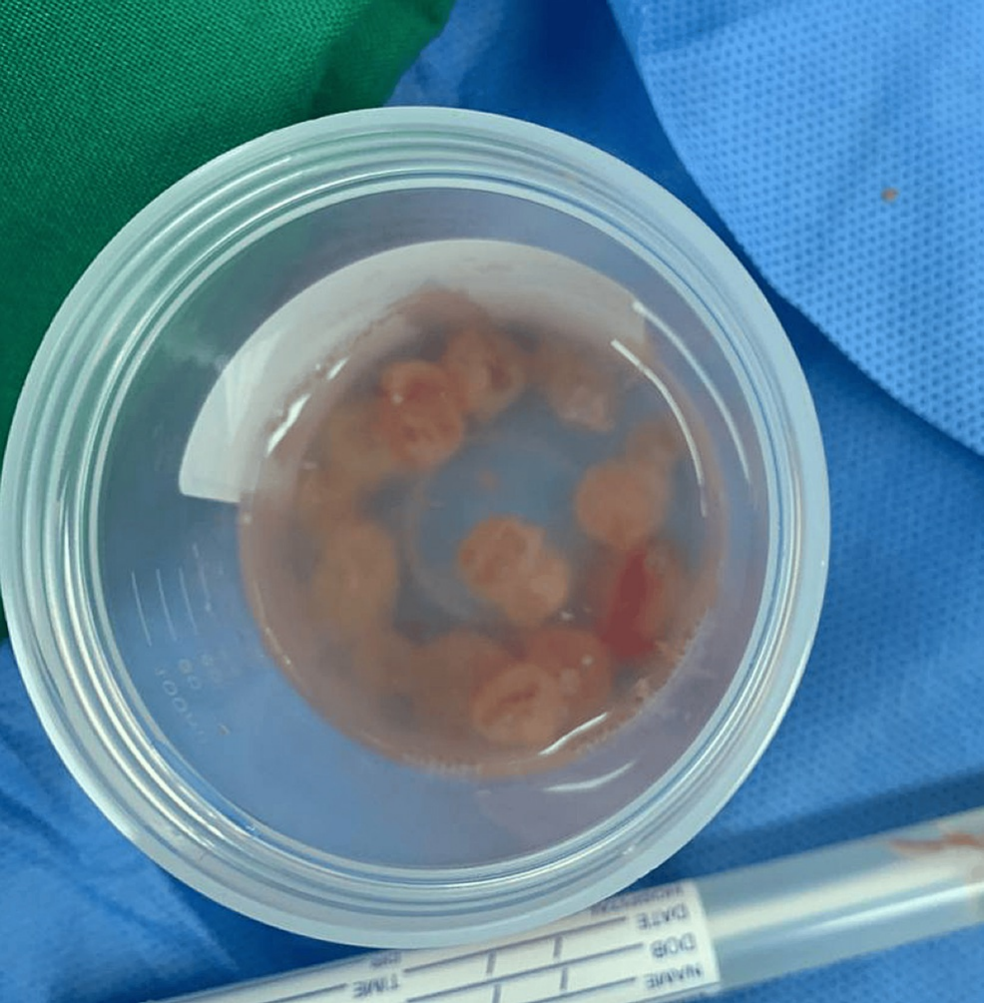
Postoperative photograph

An excision biopsy from the left thigh revealed multiple pieces of light brown fatty tissue (Figures 3, 4), inclusive of remnants of small cysts with opaque walls, measuring together 4.5 x 4 x 1.3 cm. Microscopically, the fatty tissue showed mature fat cells and acute inflammatory reactions with exudate. There were also multiple fragments of laminated acellular cyst walls surrounded by acute inflammatory cells, predominantly neutrophil cells, but no residual germinal membrane or protoscolices were observed attached to the membrane. This picture is consistent with an infected HC, and there were no signs of malignancy in the examined specimen.

Postoperatively, the patient was commenced on albendazole therapy for three months. After a stable recovery, she was discharged on the fourth postoperative day and was scheduled for a follow-up consultation after 10 days. Fortunately, she did not exhibit any local or systemic complications post surgery.

## Discussion

Hydatid disease (HD) of the musculoskeletal system represents an infrequent yet severe manifestation, predominantly secondary to the hematogenous dissemination of Echinococcus cysts from primary sites, most commonly the hepatic and pulmonary regions [1]. It is exceedingly rare for muscular hydatidosis to be the primary presentation. Clinically, these echinococcal cystic structures may remain latent for extensive durations. Their clinical significance becomes apparent predominantly when they impinge upon neurovascular entities. Such clinical presentations are accentuated in scenarios of engagement in deep muscle compartments or more prevalently when myopenic alterations and tissue attenuation precipitate pathological changes in the compromised muscular regions [11]. It is noteworthy to mention that when these cysts attain considerable dimensions, particularly when they have primary or secondary affiliations with other soft tissues, discernible swelling might be a salient clinical feature, as exemplified in the described case, which is an infected HC of the thigh muscle.

Humans serve as incidental hosts, whereas animals such as dogs, wolves, and foxes act as the primary hosts. Infection in humans can occur through ingestion of tainted food or water or by direct interaction with these animals, as evidenced by our patient who encountered a domestic cat [4].

Predominantly, the lungs and liver are the primary organs impacted by the disease. However, there have been sporadic reports highlighting the disease's manifestation in diverse anatomical structures such as the heart, bones, spleen, kidneys, brain, and eyes. Remarkably, the musculoskeletal system's involvement is an uncommon phenomenon, constituting less than 1% of all diagnosed HC cases [12,13].

It is essential to diagnose an HC preoperatively. The sensitivity of serological tests varies by type of HD; it is more sensitive to hepatic hydatidosis. Of the patients, 90% show a positive result, whereas the result may differ if the HD is in other organs. Imaging modalities can be more beneficial in diagnosing HD. Ultrasound plays a role in detecting atypical cases of HD, and sensitivity and specificity reach 100%. The location, number, and size of the cyst, as well as its relationship to surrounding structures, can all be more obvious and clearly seen on a CT scan. Though MRI is considered a diagnostic tool for identifying muscular HD, it provides extensive information about the cyst [14]. In the present case, the ultrasound’s findings showed multiple daughter cysts present in the back of the thigh. The confirmation of the diagnosis was through the MRI, which revealed a well-defined, encapsulated cystic lesion.

There are many factors involved in determining the treatment of HD, including the severity of the symptoms, the location of the cyst, and the presence of the complication [15]. Complete surgical removal is a suggestive treatment for muscular HCs. The surgeon should be careful not to cut or open the cavity to avoid the escape of the cyst's contents and to minimize the risk of recurrence. The use of anthelminthic drugs postoperatively may contribute to reducing the rate of recurrence [15,16].

## Conclusions

HC is an infrequent zoonotic disease often mimicking other clinical conditions, making its diagnosis intricate and multidimensional. The presentation of an HC in the thigh is a noteworthy contribution to medical studies because of its unusual manifestation in muscle tissue, a site not frequently linked with echinococcosis. The previous oversight in diagnosis highlights the intricacies in detecting the disease, particularly in non-standard locations, advocating for increased clinical awareness. Additionally, the patient's interaction with a domestic cat, combined with the effective surgical and medicinal treatment, provides a vital understanding of the potential transmission methods, associated risks, and therapeutic approaches for muscular hydatidosis, especially in areas where the disease is prevalent.
